# Robust Occupant Behavior Recognition via Multimodal Sequence Modeling: A Comparative Study for In-Vehicle Monitoring Systems

**DOI:** 10.3390/s25206323

**Published:** 2025-10-13

**Authors:** Jisu Kim, Byoung-Keon D. Park

**Affiliations:** 1 College of Engineering, University of Nebraska-Lincoln, Lincoln, NE 68588, USA; 2 University of Michigan Transportation Research Institute, Ann Arbor, MI 48109, USA; keonpark@umich.edu

**Keywords:** occupant behavior recognition, multimodal learning, 2D pose, gaze estimation, facial movement, temporal modeling, LSTM, MLP, Transformer, sequence classification, occupant monitoring

## Abstract

Understanding occupant behavior is critical for enhancing safety and situational awareness in intelligent transportation systems. This study investigates multimodal occupant behavior recognition using sequential inputs extracted from 2D pose, 2D gaze, and facial movements. We conduct a comprehensive comparative study of three distinct architectural paradigms: a static Multi-Layer Perceptron (MLP), a recurrent Long Short-Term Memory (LSTM) network, and an attention-based Transformer encoder. All experiments are performed on the large-scale Occupant Behavior Classification (OBC) dataset, which contains approximately 2.1 million frames across 79 behavior classes collected in a controlled, simulated environment. Our results demonstrate that temporal models significantly outperform the static baseline. The Transformer model, in particular, emerges as the superior architecture, achieving a state-of-the-art Macro F1 score of 0.9570 with a configuration of a 50-frame span and a step size of 10. Furthermore, our analysis reveals that the Transformer provides an excellent balance between high performance and computational efficiency. These findings demonstrate the superiority of attention-based temporal modeling with multimodal fusion and provide a practical framework for developing robust and efficient in-vehicle occupant monitoring systems. Implementation code and supplementary resources are available (see Data Availability Statement).

## 1. Introduction

Occupant behavior recognition has emerged as a crucial component of intelligent transportation systems, enabling real-time monitoring to enhance road safety and situational awareness. Traditional approaches often rely on single-modality visual cues and static frame-level classifiers, which struggle with the subtle, temporally dependent patterns found in complex, simulated driving environments. Moreover, a single feature type is often insufficient to capture the diverse range of behaviors, from gross body movements to fine-grained facial expressions.

Recent advancements in multimodal learning and temporal modeling have shown promise in addressing these limitations. By combining complementary cues such as body pose, gaze, and facial movements, a more holistic understanding of occupant behavior can be achieved. Temporal models, like LSTMs, can further exploit sequential dependencies to distinguish between visually similar yet temporally distinct actions. More recently, attention-based architectures such as the Transformer have demonstrated state-of-the-art performance in various sequence modeling tasks, offering an alternative approach to capturing long-range dependencies.

To address these challenges, this paper presents a lightweight and modular framework for occupant behavior recognition that leverages temporal modeling of multi-feature inputs. Our approach fuses three complementary modalities—2D pose, 2D gaze, and facial movement (FM)—into fixed-length sequences, which are then classified using three distinct architectures: a static Multi-Layer Perceptron (MLP), a recurrent Long Short-Term Memory (LSTM) network, and an attention-based Transformer encoder. We conduct a comprehensive evaluation on the large-scale Occupant Behavior Classification (OBC) dataset, and our main contributions are as follows:

- A multimodal occupant behavior recognition pipeline that integrates 2D pose, 2D gaze, and facial movement (FM) features using an early fusion strategy.
- A comparative analysis of static (MLP), recurrent (LSTM), and attention-based (Transformer) classification models, highlighting the benefits of temporal modeling for complex behavior recognition.
- An extensive ablation study on the effects of feature combinations, sequence lengths, and frame sampling strategies, providing insights into optimal design choices for in-vehicle monitoring systems.
- A lightweight and computationally efficient design suitable for practical deployment, supported by performance and inference cost evaluations.

Through these contributions, this work underscores the importance of multimodal fusion and temporal modeling for occupant behavior recognition, offering practical guidelines for the development of robust occupant monitoring systems for in-vehicle applications.

## 2. Related Work

### 2.1. Pose Estimation for Occupant Behavior

Accurate pose estimation is essential for capturing body dynamics during driving. Recent YOLO-based frameworks have demonstrated real-time, high-accuracy keypoint detection by integrating object detection and pose estimation into a unified pipeline. YOLO-Pose extends the YOLO architecture for multi-person 2D pose estimation, jointly predicting bounding boxes and keypoints in a single stage, achieving state-of-the-art performance on large-scale benchmarks [1]. Building on this, YOLOv8-PoseBoost incorporates channel attention modules, multi-scale detection heads, and cross-level feature fusion to improve small-target detection in complex environments [2]. These advances provide a robust foundation for extracting spatial cues in occupant monitoring systems.

### 2.2. Gaze Estimation

Gaze estimation is a key indicator of occupant attention and situational awareness. UniGaze [3] proposes a universal gaze estimation framework trained on large-scale, in-the-wild face datasets using masked autoencoder (MAE) [4] pre-training with a Vision Transformer backbone. This approach improves cross-domain generalization under both leave-one-dataset-out and joint-dataset evaluation protocols, making it suitable for deployment in diverse and unconstrained driving scenarios.

### 2.3. Facial Movement Modeling

Subtle facial movements can provide critical clues for identifying occupant states, such as Inattention or drowsiness. The FMAE-IAT framework [5] leverages MAE pre-training on the large-scale Face9M dataset, combined with identity adversarial training to avoid identity-dependent biases. It achieves state-of-the-art performance on action unit detection benchmarks such as BP4D [6], BP4D+ [7], and DISFA [8], highlighting its capacity to capture fine-grained facial behavior.

### 2.4. AI-Based In-Vehicle Occupant Behavior Recognition

AI-based behavior recognition is a cornerstone of modern in-vehicle occupant monitoring systems. A significant body of research has focused on driver-centric applications, leveraging machine learning and deep learning to enhance safety. Convolutional Neural Networks (CNNs), in particular, have been widely adopted for detecting driver distraction. For instance, Xing et al. (2019) [9] utilized models like AlexNet and GoogLeNet to classify seven driver activities, achieving up to 91.4% accuracy in distinguishing distracted from normal driving. Similarly, Valeriano et al. (2018) [10] recognized 10 types of distracted behaviors with 97% accuracy using a ResNet-based model. Beyond deep learning, traditional methods like Support Vector Machines (SVMs) and Decision Trees have also proven effective. Costa et al. (2019) [11] reached 89–93% accuracy in detecting driver fatigue and distraction, while Kumar and Patra (2018) [12] achieved 95.58% sensitivity in drowsiness detection using SVMs with facial features.

More recent works have adopted multimodal approaches, integrating data from RGB, depth, and infrared sensors to capture a richer representation of behavior. Ortega et al. (2020) [13] demonstrated a system that monitors distraction, drowsiness, gaze, and hand–wheel interactions, reporting performance exceeding 90%. While these foundational studies primarily target the driver for safety-critical alerts, their methodologies are broadly applicable to understanding the behaviors of all vehicle occupants, paving the way for more holistic in-cabin monitoring systems. Alongside these, attention-based models like the Transformer [14], originally developed for natural language processing, are increasingly being adapted for time-series and sequence modeling tasks due to their proficiency in capturing long-range dependencies.

### 2.5. Summary and Positioning

Previous studies have successfully established methodologies for classifying specific, often safety-critical, occupant behaviors within a limited range of 7–10 categories using techniques like CNNs and SVMs [9,10,11]. However, this focus on the driver often overlooks the broader spectrum of general occupant behaviors, and many studies do not systematically compare different feature sets and temporal modeling configurations.

In contrast, our work addresses these gaps by proposing a lightweight pipeline designed for comprehensive occupant behavior recognition. We leverage state-of-the-art pre-trained models—YOLOv8-Pose, UniGaze, and FMAE-IAT—as efficient feature extractors for three complementary cues: 2D pose, 2D gaze, and facial movement. Crucially, our work is distinguished by its validation on the large-scale Occupant Behavior Classification (OBC) dataset, which encompasses 79 diverse occupant behavior classes, moving far beyond driver-specific tasks. We conduct an extensive ablation study to systematically compare three distinct architectural paradigms, a static model (MLP), a recurrent model (LSTM), and an attention-based model (Transformer), and analyze the impact of sequence length and frame sampling strategies. This positions our work at the intersection of multimodal fusion and temporal modeling, providing a robust framework and practical insights for developing next-generation in-vehicle occupant monitoring systems.

## 3. Methodology

**Figure 1:**
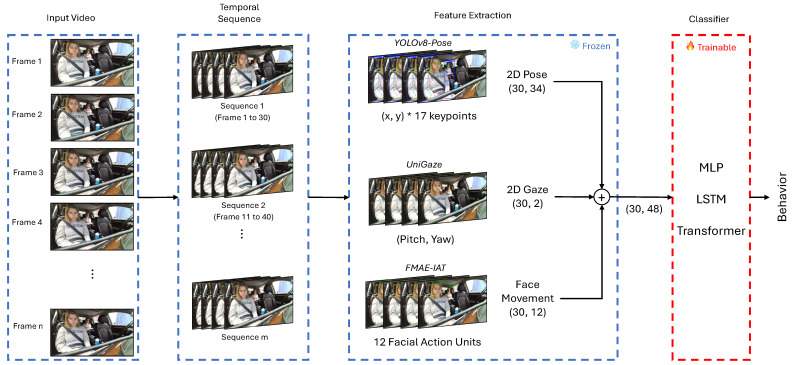
Overview of the proposed occupant behavior recognition pipeline, now including the Transformer model as a classifier.

To address the challenges of recognizing complex, temporally dependent occupant behaviors, we designed a lightweight and modular recognition pipeline. Our approach prioritizes both high accuracy through multimodal fusion and computational efficiency by freezing feature extractors. As illustrated in Figure 1, the pipeline is divided into three main stages:

- Feature Extraction: For each input frame, we extract three types of features—2D pose, 2D gaze, and facial movement (FM). Pre-trained models are used to extract these features, and to improve computational efficiency, the feature extractors are frozen during training.
- Fusion and Sequence Construction: The extracted features from each modality are concatenated to form a unified feature vector per frame. Then, consecutive frames are grouped into sequences based on a specified number of frames and step size.
- Temporal Classification: The constructed sequences are fed into a lightweight classifier. We compare three distinct architectures: a static MLP, a recurrent LSTM, and an attention-based Transformer. Only the classifier is trainable, keeping the rest of the pipeline fixed.

This modular design allows easy experimentation with different combinations of input features, sequence lengths, and model architectures, facilitating both ablation and computational cost analysis.

### 3.1. Multi-Feature Fusion

To construct a comprehensive representation of occupant behavior, we fuse three complementary modalities: 2D pose, 2D gaze, and facial movement (FM). Each feature type captures a different aspect of occupant behavior: pose encodes gross body movement, gaze reflects visual attention, and FM captures subtle expressions related to the occupant’s state (e.g., drowsiness or inattention). Each modality is processed by a specialized, pre-trained feature extractor chosen for its state-of-the-art performance and efficiency, as discussed in Section 2.

- **Two-dimensional Pose**: We employ YOLOv8-Pose [15], selected for its high accuracy and real-time keypoint detection capabilities crucial for in-vehicle monitoring.
- **Two-dimensional Gaze**: We use UniGaze [3], which offers robust cross-domain generalization, making it suitable for diverse and unconstrained driving scenarios.
- **Facial Movement**: We utilize FMAE-IAT [5] to extract a 12-dimensional vector of Facial Action Units (AUs). The process involves detecting and cropping the occupant’s face, resizing it, and feeding it into the frozen FMAE-IAT feature extractor, which directly outputs the 12-dimensional AU intensity vector.

Once extracted, the features from each modality are concatenated along the channel axis for each frame. This early fusion strategy allows the temporal model to learn from a unified representation that incorporates information across all modalities. By design, these feature extraction modules are frozen during training to maintain a lightweight pipeline and ensure computational efficiency.

### 3.2. Temporal Sequence Modeling

**Figure 2:**
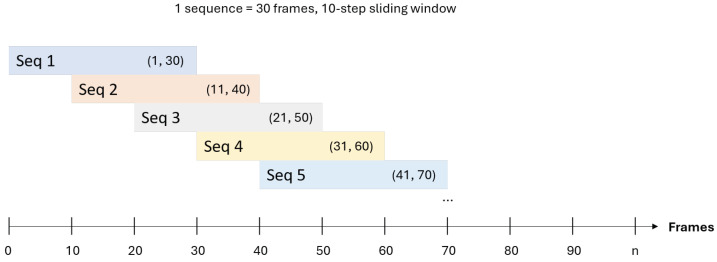
Illustration of temporal sequence sampling with overlapping windows.

Occupant behaviors are inherently temporal phenomena. To effectively model these dynamics while managing computational load, we transform the continuous video data into discrete sequences using a two-stage sampling process governed by three key hyperparameters, as illustrated in Figure 2.

First, we define a sequence span ($L_{span}$), which is the total duration of the temporal window from the raw video. Second, from within this span, we downsample a fixed number of frame samples ($L_{samples}$). These frames are selected at a uniform interval to form the final input sequence.

Finally, the step size (*S*) determines the offset by which this entire sequence span window is moved to create the next overlapping sequence. A single ground-truth label is assigned to each final sequence by taking the majority vote of the frame-level labels within its span.

### 3.3. Classifier Architectures

For classifying the fused feature sequences, we implemented and compared three architectures representing different modeling paradigms: a static model (MLP), a recurrent model (LSTM), and an attention-based model (Transformer). Our design focuses on keeping these classifiers lightweight while freezing the upstream feature extractors, which is critical for practical deployment. The detailed architectural parameters for each model are summarized in Table 1.

#### 3.3.1. Multi-Layer Perceptron (MLP)

The MLP serves as our static baseline. It processes a sequence by flattening all temporal features into a single large vector, thus ignoring explicit temporal ordering. Our implementation consists of four fully connected layers with ReLU activations and batch normalization, which progressively reduce the feature dimension before a final classification layer.

#### 3.3.2. Long Short-Term Memory (LSTM)

As a representative recurrent model, the LSTM is chosen for its ability to model temporal dependencies by processing sequences step by step and maintaining an internal memory state. We use a three-layer unidirectional LSTM, where the mean-pooled output of the final time step’s hidden state is passed through a layer normalization step before being used for classification.

#### 3.3.3. Transformer

To represent attention-based models, we use a Transformer encoder architecture. The model first projects the input features into a higher-dimensional space and adds sinusoidal positional encodings to retain sequence order. The data is then processed by a stack of four multi-head self-attention layers, which allows the model to weigh the importance of all frames in the sequence simultaneously. The final classification is made from the mean-pooled and layer-normalized output of the encoder.

## 4. Experiments

In this section, we describe the dataset used in our study, the evaluation metrics employed, and the implementation and training details and provide a comprehensive analysis of our results, including an ablation study to examine the contribution of each component.

### 4.1. Dataset

For this study, we utilized the Occupant Behavior Classification (OBC) dataset. This dataset was originally collected at the University of Michigan Transportation Research Institute (UMTRI) to investigate occupant behaviors across different levels of simulated vehicle automation (protocol approved by the UMTRI Institutional Review Board: HUM00162942). The dataset is not publicly available due to privacy protection considerations. The data collection included 42 licensed drivers (21 men and 21 women) with a broad range of anthropometric characteristics and ages from 18 to 59 years. All participants were recorded in a stationary 2018 Hyundai Genesis G90 sedan equipped with two Microsoft Azure Kinect sensors mounted near the A-pillars to capture both front seats.

The dataset contains approximately 2.1 million frames captured at 10 frames per second with a resolution of 1280×720. It covers 79 distinct occupant behavior classes, which were elicited by asking participants to perform a series of scripted tasks. To elicit naturalistic-style behavior, participants were instructed to perform these tasks as they normally would in a real moving vehicle and to find postures they would consider comfortable for a long ride. These tasks were performed under three simulated automation levels: Manual (MN), Fully Automated (FA), and Semi-Automated (SA). For the MN and SA sessions, the participant was seated in the driver’s seat, while for the FA session, they were moved to the passenger’s seat to reflect a non-driving role. The data includes synchronized video from two front-facing camera views, one positioned in front of the driver seat and the other in front of the passenger seat. The OBC dataset captures a variety of controlled driving conditions, including scenarios with a single driver as well as those with passengers seated in the back. Each frame is annotated with a single occupant behavior class.

For our experiments, the dataset was split into training (80%, 1.68 M frames), validation (10%, 210 K frames), and testing (10%, 210 K frames) subsets. The full list of behavior classes is provided in Appendix A. It is important to note the constraints of the data collection environment. The experiments were conducted in a stationary vehicle with a locked steering wheel, and some seat adjustment controls were deactivated to standardize conditions. Behaviors were elicited via scripted prompts from an investigator, which may differ from fully spontaneous actions in an on-road driving context.

### 4.2. Evaluation Metrics

To evaluate the performance of occupant behavior recognition models, we adopt five widely used metrics for multi-class classification: accuracy, Balanced Accuracy, Macro F1, Weighted F1, and the confusion matrix. Accuracy measures the overall proportion of correctly classified instances:

(1) $$\mathrm{Accuracy}=\frac{1}{N}\sum_{i=1}^{N}1\left({\hat{y}}_{i}=y_{i}\right)$$

where *N* is the total number of instances, $y_{i}$ is the ground-truth label, ${\hat{y}}_{i}$ is the predicted label, and 1(·) is the indicator function. Balanced Accuracy computes the average recall over all *C* classes, mitigating the impact of class imbalance:

(2) $$\mathrm{Balanced}\;\mathrm{Accuracy}=\frac{1}{C}\sum_{c=1}^{C}\frac{TP_{c}}{TP_{c}+FN_{c}}$$

where $TP_{c}$ and $FN_{c}$ denote the true positives and false negatives for class *c*. Macro F1 is the unweighted average of per-class F1-scores:

(3) $$\mathrm{Macro}\;F1=\frac{1}{C}\sum_{c=1}^{C}{F1}_{c}\;\mathrm{with}\;{F1}_{c}=\frac{2\;\cdot \;{\mathrm{Precision}}_{c}\;\cdot \;{\mathrm{Recall}}_{c}}{{\mathrm{Precision}}_{c}+{\mathrm{Recall}}_{c}}$$

Weighted F1 computes the F1-score per class and weights each score by the number of instances in that class:

(4) $$\mathrm{Weighted}\;F1=\sum_{c=1}^{C}\frac{n_{c}}{N}\;\cdot \;{F1}_{c}$$

where $n_{c}$ is the number of true instances of class *c*. A confusion matrix is a C×C matrix *M*, where $M_{i,j}$ denotes the number of instances of class *i* predicted as class *j*. It provides a detailed visualization of misclassifications:

$$M_{i,j}=\#\left\{\mathrm{samples}\;\mathrm{where}\;y=i\;\mathrm{and}\;\hat{y}=j\right\}$$

The full confusion matrix for all 79 classes is provided in Appendix B.

### 4.3. Experimental Setup

We trained and evaluated all three models—MLP, LSTM, and Transformer—under a consistent experimental framework to ensure a fair comparison. The architectural details of each model are described in Section 3.3. For the sequential models (LSTM and Transformer), we conducted an extensive ablation study on temporal configurations by varying the sequence span ($L_{span}$), step size (*S*), and the number of frame samples ($L_{samples}$). Each sequence was assigned a single ground-truth label based on the majority vote of its constituent frames.

All models were trained using the Adam optimizer for up to 200 epochs, employing an early stopping mechanism with a patience of 10 epochs based on the validation loss. The key training hyperparameters, such as learning rate, batch size, and dropout, are summarized for each model in Table 2. All experiments were implemented in PyTorch (version 2.7.1+cu126) and executed on a high-performance computing cluster equipped with an NVIDIA Tesla V100 GPU (Santa Clara, CA, USA).

## 5. Results

This section presents a comprehensive evaluation of our proposed framework, comparing the performance of the MLP, LSTM, and Transformer models. We analyze the results from four perspectives: the impact of input feature modalities, the effect of temporal configurations on the Transformer model, a direct comparison of model performance versus computational efficiency, and an in-depth analysis of per-class performance.

### 5.1. Input Modality Ablation Study

To understand the contribution of each visual cue, we first evaluated all three models with various combinations of 2D pose, 2D gaze, and facial movement (FM) features, using a fixed sequence length of 30 frames. As shown in Table 3, several key trends emerge. First, 2D pose is consistently the most dominant modality, providing a strong performance baseline. Second, both LSTM and Transformer significantly outperform the static MLP model across all feature combinations, underscoring the importance of temporal modeling. Third, the Transformer model generally achieves the highest performance, particularly when modalities are fused. The best overall result is achieved when all three modalities (‘Pose + Gaze + FM’) are used with the Transformer, reaching a Macro F1 of 0.8970.

### 5.2. Temporal Configuration Analysis for the Transformer

Given the strong performance of the Transformer, we conducted an extensive ablation study to analyze its sensitivity to different temporal configurations, with detailed results presented in Table 4. The results indicate that a smaller, denser step size (*S*) consistently yields better performance. For instance, with a sequence span ($L_{span}$) of 50, a step size of 10 achieves a Macro F1 of 0.9570, whereas a step size of 50 results in a score of only 0.3012. The number of frame samples ($L_{samples}$) also plays a crucial role. The highest performance was achieved with a 50-frame span and a step size of 10. Specifically, the configuration with $L_{samples}=50$ yielded the best Macro F1 score of 0.9570, while the configuration with $L_{samples}=25$ achieved the highest Balanced Accuracy of 0.9567.

### 5.3. Performance vs. Efficiency Comparison

A critical aspect for practical deployment is the trade-off between predictive performance and computational cost. We summarize this comparison in Table 5. As expected, the MLP is the most lightweight model but provides the lowest performance. While the LSTM model shows the highest peak performance (Macro F1 of 0.9931), this result stems from our initial experimental design using a frame-level data split. As detailed in our Discussion (Section 6), this approach can lead to performance inflation. In contrast, the Transformer model offers a compelling balance. Its best-performing configuration ($L_{span}=50,S=10,L_{samples}=50$) achieves a high and, crucially, more robust Macro F1 score of 0.9570. This positions the Transformer as the superior architecture, providing state-of-the-art performance within our revised framework. Furthermore, its most efficient configuration ($L_{span}=10,S=5,L_{samples}=5$) delivers a strong Macro F1 of 0.9395 with only 0.02 GFLOPs, highlighting its suitability for resource-constrained environments.

### 5.4. Per-Class Performance and Error Analysis

To gain deeper insights into the Transformer model’s behavior, we analyzed its per-class performance using its best-performing configuration, as detailed in Table 6. A notable finding is the model’s exceptionally high performance even on what are predicted to be challenging classes. The Top-5 performing classes are distinct actions like ‘Tilting sun visor’ or ‘Using laptop on armrest’. More impressively, the Bottom-5 classes, which often involve subtle motions or have low sample counts (e.g., ‘Adjusting pelvis in seat’), still achieve F1 scores near or above 0.90. This demonstrates the Transformer’s strong ability to capture discriminative features even from limited data.

**Figure 3:**
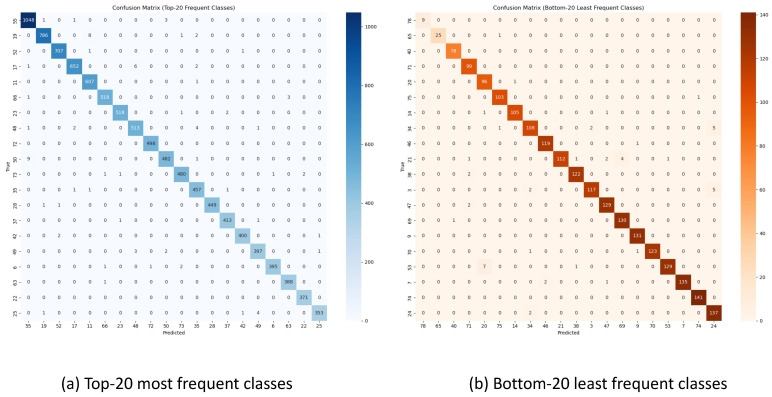
Confusion matrices for the top-20 and bottom-20 classes, generated from the best-performing Transformer model.

This high overall performance is also reflected in the confusion matrices shown in Figure 3. For the 20 most frequent classes, the matrix shows a strong diagonal, indicating few misclassifications. For instance, some notable confusion can be observed between similar fine-grained tasks, such as different types of phone use or subtle posture changes. While the bottom-20 classes show slightly more confusion, the overall performance remains robust, consistent with the findings in our per-class analysis.

## 6. Discussion

Our experimental results provide several key insights into multimodal temporal modeling for occupant behavior recognition. This section discusses the implications of our findings, focusing on the comparison between static, recurrent, and attention-based models, the role of multimodal fusion, the trade-off between performance and efficiency, and the surprising robustness of our best model.

First, our comparative analysis confirms the critical importance of temporal modeling. As shown in the input modality ablation study (Table 3), both the LSTM and Transformer architectures substantially outperform the static MLP across all feature combinations. This demonstrates that capturing the sequential nature of actions is fundamental to achieving high accuracy. Between the two temporal models, the Transformer consistently shows a competitive edge, especially with fused modalities like ‘Pose + FM’, suggesting that its self-attention mechanism is highly effective for this task.

Second, the analysis of temporal configurations for the Transformer (Table 4) reveals a clear pattern: denser, more overlapping sequences created with smaller step sizes yield superior results. However, this increased performance comes at a higher computational cost. The trade-off between performance and efficiency, summarized in Table 5, is central to our findings. The MLP is the most efficient but least accurate model. In contrast, the Transformer presents a compelling balance; it achieves high performance (Macro F1 of 0.9561) while being significantly more resource-efficient than the LSTM in terms of parameters and FLOPs. This positions the Transformer as a strong candidate for practical, resource-constrained in-vehicle systems.

Third, the per-class performance analysis of our best Transformer model (Table 6) offers further insights into its robustness. A key finding is the model’s exceptionally high F1 scores even for its “Bottom-5” classes, which remain near or above 0.90. These classes, such as ‘Adjusting pelvis in seat’, are characterized by low support counts and subtle motions. This suggests that the Transformer’s self-attention mechanism is highly effective at learning discriminative patterns even from limited examples. This is visually corroborated by the confusion matrices in Figure 3, which display a strong diagonal dominance.

Finally, our study has several key limitations. The frame-level splitting of the dataset introduces potential data leakage from two perspectives. First, it does not guarantee that the training, validation, and test sets are subject-disjoint, which presents a risk of the model learning subject-specific mannerisms (identity leakage). Second, it preserves temporal continuity across the split boundaries, meaning a sequence at the beginning of the validation set can be a direct continuation of a sequence from the training set. Both factors can inflate performance and limit conclusions about generalization. Furthermore, the OBC dataset was gathered in a stationary vehicle with scripted tasks, not in an actual on-road driving context. Generalizing these findings to unconstrained scenarios requires further validation. The model was also not evaluated under challenging conditions common in on-road driving, such as poor illumination, partial occlusions, or unscripted, extreme postures. Additionally, while our analysis provides efficiency metrics on a high-performance GPU (Table 5), we did not benchmark the model on embedded hardware, such as the NVIDIA Jetson series, which is more typical for in-vehicle applications. Although our lightweight design with frozen feature extractors is a strong candidate for such resource-constrained environments, formal validation of its real-time performance on such hardware remains a critical task for future work. Lastly, this study did not include a fairness analysis to assess potential performance biases related to demographic factors such as gender or age. Future work should investigate the model’s performance across these groups to ensure the system is equitable and reliable for all occupants. We contend that these risks are partially mitigated by our feature-based approach. Nonetheless, future research must validate this framework using a strict subject-disjoint split on datasets captured in more naturalistic on-road conditions to confirm its real-world applicability.

## 7. Conclusions

In this paper, we presented and evaluated a lightweight, modular framework for occupant behavior recognition using multimodal visual features. Our approach effectively fused 2D pose, 2D gaze, and facial movement features and utilized three distinct classifier architectures—a static MLP, a recurrent LSTM, and an attention-based Transformer—to model the temporal dynamics of 79 distinct behaviors from the OBC dataset.

Our comprehensive experiments demonstrated several key findings: (1) temporal models (LSTM and Transformer) significantly outperform static, frame-based MLP classification, confirming the importance of sequential context; (2) fusing all three modalities consistently yields the best performance for the temporal models, validating the benefits of a multimodal approach; and (3) the Transformer model achieved the best overall performance, reaching a Macro F1 score of 0.9570 with a configuration of a 50-frame span, a step size of 10, and 25 sampled frames. Furthermore, our analysis revealed that the Transformer offers a superior balance between high accuracy and computational efficiency, positioning it as a strong candidate for practical, resource-constrained systems.

Overall, this work underscores the critical importance of integrating temporal context and complementary multimodal features for robust occupant behavior recognition. The findings provide a strong foundation and practical guidelines for the development of next-generation, computationally efficient in-vehicle occupant monitoring systems, with the Transformer architecture emerging as a particularly promising solution.

## Figures and Tables

**Table 1:** Detailed architectures of the implemented classifier models. The input for sequential models is a sequence of fused feature vectors, while the MLP uses a flattened version of this sequence.

Parameter	MLP	LSTM	Transformer Encoder
Input Dimension	$\left(L_{samples}\times 48\right)$	$L_{samples}\times 48$	$L_{samples}\times 48$
Layer Configuration	Input→256→128→64→79	-	-
Number of Layers	4 Fully-Connected	3 Layers	4 Encoder Layers
Hidden Dimension	-	256	256
Number of Heads	-	-	8
Activation Function	ReLU	Tanh	ReLU
Normalization	BatchNorm1d	LayerNorm	LayerNorm
Output Dimension	79	79	79

**Table 2:** Key hyperparameters used for training the MLP, LSTM, and Transformer models.

Hyperparameter	MLP	LSTM	Transformer
Learning Rate	$1\times 10^{-3}$	$1\times 10^{-3}$	$1\times 10^{-4}$
Optimizer	Adam	Adam	Adam
Batch Size	256	256	128
Dropout	0.0	0.0	0.2
Epochs	200	200	200
Early Stopping Patience	10	10	10

**Table 3:** Test results on the OBC dataset using different feature combinations and models. Input sequences use 30 frames with a step size of 10. The upward arrow (↑) next to each metric indicates that higher values are better. For each metric, the best result among models for a given feature set is highlighted in **bold**.

Features	Model	Accuracy ↑	Bal. Acc. ↑	Weighted F1 ↑	Macro F1 ↑
Pose	MLP	0.6880	0.6278	0.6836	0.6358
	LSTM	0.9027	**0.8784**	0.9025	**0.8784**
	Transformer	**0.9084**	0.8780	**0.9084**	0.8759
Gaze	MLP	0.0701	0.0320	0.0273	0.0150
	LSTM	0.1387	0.1106	0.1275	0.1145
	Transformer	**0.1701**	**0.1331**	**0.1576**	**0.1338**
FM	MLP	0.3285	0.2408	0.2984	0.2386
	LSTM	0.5928	0.5385	0.5889	0.5425
	Transformer	**0.7635**	**0.7134**	**0.7623**	**0.7158**
Pose + Gaze	MLP	0.6875	0.6308	0.6833	0.6375
	LSTM	0.9080	**0.8853**	0.9078	**0.8875**
	Transformer	**0.9084**	0.8796	**0.9084**	0.8785
Pose + FM	MLP	0.7202	0.6609	0.7167	0.6668
	LSTM	0.9072	0.8812	0.9069	0.8838
	Transformer	**0.9349**	**0.9069**	**0.9348**	**0.9081**
Gaze + FM	MLP	0.3442	0.2653	0.3205	0.2663
	LSTM	0.5708	0.5277	0.5678	0.5306
	Transformer	**0.7094**	**0.6584**	**0.7083**	**0.6611**
Pose + Gaze + FM	MLP	0.7235	0.6651	0.7201	0.6705
	LSTM	0.9185	0.8913	0.9183	0.8941
	Transformer	**0.9248**	**0.8996**	**0.9249**	**0.8970**

**Table 4:** Comprehensive performance analysis of the Transformer model across varying temporal configurations. The overall best-performing configuration is highlighted in **bold**.

Configuration	Performance Metrics			
($L_{\mathrm{span}}$, S, $L_{\mathrm{samples}}$)	Accuracy	Bal. Acc.	Weighted F1	Macro F1
(10, 5, 5)	0.9531	0.9390	0.9530	0.9395
(10, 5, 10)	0.9468	0.9317	0.9468	0.9297
(10, 10, 5)	0.8472	0.8061	0.8466	0.8090
(10, 10, 10)	0.8559	0.8130	0.8555	0.8144
(20, 10, 10)	0.8753	0.8433	0.8752	0.8439
(20, 10, 20)	0.9148	0.8863	0.9147	0.8854
(20, 20, 10)	0.6881	0.6245	0.6870	0.6252
(20, 20, 20)	0.6429	0.5702	0.6404	0.5708
(30, 10, 15)	0.9306	0.9090	0.9307	0.9080
(30, 10, 30)	0.9305	0.9075	0.9305	0.9066
(30, 15, 15)	0.8489	0.8071	0.8488	0.8078
(30, 15, 30)	0.8109	0.7626	0.8104	0.7653
(30, 30, 15)	0.5802	0.5140	0.5763	0.5139
(30, 30, 30)	0.5217	0.4587	0.5164	0.4609
(40, 10, 20)	0.9523	0.9360	0.9524	0.9360
(40, 10, 40)	0.9438	0.9270	0.9438	0.9271
(40, 20, 20)	0.7485	0.6982	0.7483	0.6996
(40, 20, 40)	0.7340	0.6842	0.7336	0.6858
(40, 40, 20)	0.4688	0.4275	0.4607	0.4242
(40, 40, 40)	0.4104	0.3524	0.3955	0.3511
(50, 10, 25)	0.9676	0.9567	0.9676	0.9561
**(50, 10, 50)**	**0.9675**	**0.9561**	**0.9675**	**0.9570**
(50, 25, 25)	0.6441	0.6069	0.6410	0.6010
(50, 25, 50)	0.6671	0.6204	0.6645	0.6227
(50, 50, 25)	0.4178	0.3645	0.4060	0.3632
(50, 50, 50)	0.3516	0.3000	0.3364	0.3012

**Table 5:** Computational efficiency and performance comparison of the models. The Transformer is evaluated on its best-performing and most resource-efficient configurations. The best overall configuration balancing performance and efficiency is highlighted in **bold**.

Model	Configuration	Macro F1	Params (M)	GFLOPs	Time (ms)	F1/GFLOPs
MLP	Frame-level	0.6705	0.06	<0.001	0.08	-
LSTM (Low-Cost)	(10, 10, 5)	0.6601	1.39	0.01	0.17	66.01
LSTM (High-Perf.)	(40, 10, 40)	0.9931	1.39	0.05	0.44	19.86
Transformer (Efficient)	(10, 5, 5)	0.9395	4.24	0.02	0.33	46.97
**Transformer (Best-Perf.)**	**(50, 10, 50)**	**0.9570**	**4.24**	**0.21**	**0.34**	**4.55**

**Table 6:** In-depth analysis of the Transformer model’s per-class performance, showing the Top-5 and Bottom-5 classes based on their F1 scores. Support indicates the number of test samples for each class. Full behavior descriptions are available in Appendix A.

Group	Class ID	Behavior Description (Summarized)	F1 Score	Support
Top-5	78	Tilting sun visor	1.0000	9
	13	Using laptop on armrest	0.9916	239
	11	Repositioning with laptop	0.9902	608
	17	Finding new resting posture	0.9901	661
	55	Repositioning with phone	0.9901	1056
Bottom-5	70	Removing/donning seat belt	0.9077	137
	12	Reaching to passenger floor	0.9074	159
	20	Adjusting vent settings	0.9057	103
	40	Adjusting pelvis in seat	0.8966	90
	34	Using visor mirror	0.8963	123

**Figure A1:**
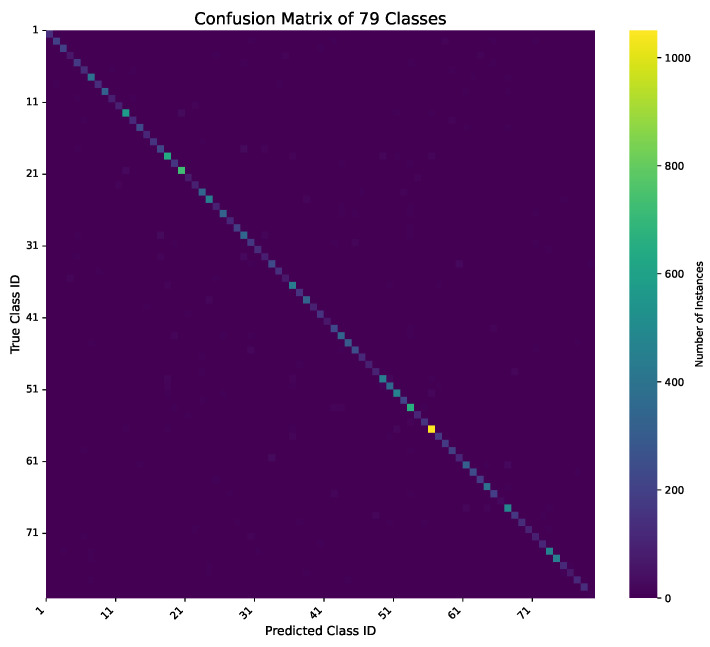
Heatmap visualization of the 79×79 confusion matrix. The color intensity corresponds to the number of instances, showing where the model most often confuses classes.

## Data Availability

The raw, participant-level data that underpin the results of this study are not publicly available because they contain personally identifiable information (PII) and their release would risk participant privacy. Implementation code, trained model weights (where applicable), and supplementary, non-identifiable materials (example inputs, synthetic samples, and evaluation scripts) are publicly available at the authors’ GitHub repository: https://github.com/wltnkim/UMTRI_Occupant_Behavior, accessed on 9 October 2025. Requests for access to de-identified or restricted data may be considered on a case-by-case basis and will require approval from the Institutional Review Board (IRB) and a signed data use agreement.

## Appendix A. List of Behavior Classes

The complete list of the 79 classes from the Occupant Behavior Classification (OBC) dataset is detailed in Table A1 and Table A2. Each class represents a unique occupant behavior performed under a specific, simulated driving condition. A more comprehensive description of the dataset’s composition and data collection protocol is provided in Section 4.1.

**Table A1:** A detailed list of the 79 behavior classes in the OBC dataset (Part 1 of 2).

ID	Driving Mode	Behavior Category	Detailed Description
0	Fully Automated	Posture Change	Change head positions: Use the headrest or use a hand to support the head.
1	Manual	Head Range of Motion	Rotation (Left/Right): Turn head as far as possible to the left/right while still being able to drive.
2	Fully Automated	Reaching	Reaching to floor: Reach to the floor by the right and left foot to pick something up.
3	Manual	Non-Driving Task	Take a drink from the water bottle and then return it to the cupholder.
4	Manual	Non-Driving Task	Talk over right shoulder to a passenger in the rear seat.
5	Manual	Reaching	Reach to the glove box (or as far right as possible on the dash).
6	Fully Automated	Phone Use	Select other body postures for using a phone (seat adjustment allowed).
7	Manual	Head Range of Motion	Extension: Tip head backward, rotating face to the ceiling as far as possible while driving.
8	Fully Automated	Laptop Use	Reposition legs to hold the laptop differently.
9	Manual	Reaching	Reach to the center of the passenger seat cushion.
10	Fully Automated	Reaching	Reach to the floor directly in front of the driver seat.
11	Semi-Automated	Laptop Use	In auto-mode, select another laptop location and posture and then type and read for 10 seconds.
12	Manual	Reaching	Reach to the floor directly in front of the passenger seat.
13	Fully Automated	Laptop Use	Place the laptop on the center armrest to use it.
14	Fully Automated	Non-Driving Task	Talk over left shoulder to a passenger in the rear seat.
15	Fully Automated	Vehicle Interaction	Remove and don the seat belt.
16	Manual	Vehicle Interaction	Open and then close the sunglasses compartment above the center mirror.
17	Semi-Automated	Sleep/Resting	In auto-mode, use armrest, door, or seat contours to find other comfortable resting postures.
18	Manual	Vehicle Interaction	Change the vent settings.
19	Semi-Automated	Laptop Use	In auto-mode, use armrest or door to find other postures for using the laptop.
20	Fully Automated	Vehicle Interaction	Change the vent settings.
21	Fully Automated	Reaching	Reach to the center of the driver seat cushion.
22	Fully Automated	Non-Driving Task	Use phone to make a call (using right hand, left hand, and speaker).
23	Fully Automated	Sleep/Resting	Select other body postures for sleeping/resting (seat adjustment allowed).
24	Manual	Non-Driving Task	Use phone to make a call (using right hand, left hand, and speaker).
25	Semi-Automated	Driving Task	Transition from manual to auto-mode, check mirrors, and then perform a takeover request.
26	Manual	Reaching	Reach behind the passenger seat (as a specific reaching task).
27	Manual	Posture Change	Change head positions: Use the headrest or use a hand to support the head.
28	Manual	Vehicle Interaction	Pretend to press one of the seat position memory buttons on the door by the left knee.
29	Manual	Driving Task	Check right/left blind spot and pretend to change lanes.
30	Fully Automated	Posture Change	Use armrests (center or door) to adjust position.
31	Manual	Non-Driving Task	Use the mirror on the back of the visor to look at your face (as a primary task).
32	Manual	Reaching	Reach behind the passenger seat (during a general posture change).
33	Manual	Head Range of Motion	Flexion: Tip chin to chest as far as possible while driving.
34	Manual	Non-Driving Task	Use the mirror on the back of the visor to look at your face (during sun visor adjustment).
35	Semi-Automated	Riding Task	In auto-mode, select another comfortable body posture (seat adjustment allowed).
36	Manual	Torso Range of Motion	Slouch: Push body back and slide hips forward as far as possible while driving.
37	Fully Automated	Sleep/Resting	Recline seat more, lean on vehicle side, or rest head on hand.
38	Fully Automated	Reaching	Reach to the area of the steering wheel.
39	Fully Automated	Vehicle Interaction	Pretend to press one of the seat position memory buttons on the door near the right knee.

**Table A2:** A detailed list of the 79 behavior classes in the OBC dataset (Part 2 of 2).

ID	Driving Mode	Behavior Category	Detailed Description
40	Fully Automated	Posture Change	Adjust pelvis in the seat.
41	Manual	Torso Range of Motion	Rotation (Left/Right): Twist body as much as possible to the left/right while driving.
42	Manual	Driving Task	Try out other hand positions on the steering wheel (right hand only, left hand only).
43	Fully Automated	Phone Use	Move the phone to other positions (lower, higher, to the side) and continue to use it.
44	Manual	Driving Task	Check mirrors (center, left, right), moving head to see more of the field of view.
45	Fully Automated	Non-Driving Task	Take a drink from the water bottle and then return it to the cupholder.
46	Manual	Torso Range of Motion	Flexion: Tilt body forward toward the steering wheel as far as possible.
47	Fully Automated	Non-Driving Task	Use the mirror on the back of the visor to look at your face.
48	Semi-Automated	Sleep/Resting	In auto-mode, perform takeover, return to resting, and then select another resting posture.
49	Semi-Automated	Sleep/Resting	In auto-mode, receive a “takeover in 2 miles” warning, adjust seat to prepare, and then takeover.
50	Semi-Automated	Phone Use	While using phone in auto-mode, perform a takeover and then return to preferred position.
51	Fully Automated	Non-Driving Task	Use phone to text or to look at a map.
52	Manual	Posture Change	Select other body postures for driving (seat adjustment allowed).
53	Fully Automated	Vehicle Interaction	Change the fan speed or temperature using the controls on the dash.
54	Manual	Posture Change	Use armrests (center or door) to adjust position.
55	Semi-Automated	Phone Use	In auto-mode, find a new comfortable position while using phone (seat adjustment allowed).
56	Semi-Automated	Riding Task	In auto-mode, check mirrors and then perform a takeover request.
57	Manual	Head Range of Motion	Lateral Bend (Left/Right): Tilt head to the left/right, ear toward shoulder.
58	Manual	Reaching	Reach to the floor by the right and left foot to pick something up.
59	Manual	Vehicle Interaction	Change the fan speed or temperature using the controls on the dash.
60	Fully Automated	Laptop Use	Move the laptop to other resting positions and continue typing/reading.
61	Fully Automated	Phone Use	Try holding the phone at different locations such as lower, higher, or to the side.
62	Manual	Driving Task	Simulate stopping the car, shifting to park, and reversing for 3 s.
63	Fully Automated	Laptop Use	Use a laptop browser to search a topic, including typing and reading.
64	Manual	Torso Range of Motion	Lateral Bend (Left/Right): Tilt body as far as possible to the left/right while driving.
65	Fully Automated	Standard Posture	Standard posture: Seated full rear, feet forward on heels, hands on lap, looking forward.
66	Fully Automated	Laptop Use	Select other body postures for using a laptop (seat adjustment allowed).
67	Fully Automated	Vehicle Interaction	Tilt sun visor down, to the side, and back up.
68	Manual	Posture Change	Adjust pelvis in the seat to be more relaxed (slouching) or more alert.
69	Fully Automated	Reaching	Reach behind the driver seat.
70	Manual	Vehicle Interaction	Remove and don the seat belt.
71	Fully Automated	Vehicle Interaction	Open and then close the sunglasses compartment by the center mirror.
72	Fully Automated	Posture Change	Select other body postures for riding as a passenger (seat adjustment allowed).
73	Fully Automated	Phone Use	Use phone for various tasks: look up number, call, text, use browser/maps, view video.
74	Fully Automated	Sleep/Resting	Change head positions: Use the headrest or use a hand to hold the head up.
75	Fully Automated	Sleep/Resting	Adjust pelvis in the seat.
76	Fully Automated	Phone Use	Rest elbow on the armrest while holding and using the phone.
77	Manual	Non-Driving Task	Use phone to text or look at a map while considering how to hold it for navigation.
78	Manual	Vehicle Interaction	Tilt sun visor down, to the side, and back up.

## Appendix B. Full Confusion Matrix

Figure A1 presents a heatmap visualization of the 79×79 confusion matrix, illustrating the model’s classification performance on the test set. The vertical axis represents the true class labels, while the horizontal axis represents the labels predicted by the model. The color intensity of each cell corresponds to the number of instances, with brighter colors along the main diagonal indicating a high number of correct predictions. Off-diagonal bright spots highlight specific classes that the model tends to confuse. The complete list of class IDs for the axes is detailed in Appendix A.
